# Nitrate reductase enzymes in alga *Chattonella subsalsa* are regulated by environmental cues at the translational and post-translational levels

**DOI:** 10.3389/fmicb.2023.1059074

**Published:** 2023-03-02

**Authors:** Yanfei Wang, Gretchen I. Johnson, Anna Postles, Kathryn J. Coyne

**Affiliations:** School of Marine Science and Policy, College of Earth, Ocean, and Environment, University of Delaware, Lewes, DE, United States

**Keywords:** *Chattonella subsalsa*, nitrate reductase, 14-3-3 binding, harmful algal blooms, *Heterosigma akashiwo*, truncated 2/2 hemoglobin, NR3, phosphorylation

## Abstract

Nitrate reductase (NR) catalyzes the rate-limiting step in nitrate assimilation. Plant and algal NRs have a highly conserved domain architecture but differ in regulation. In plants, NR activity is regulated by reversible phosphorylation and subsequent binding of 14-3-3 proteins at a conserved serine residue. Algal NRs typically lack 14-3-3 binding motifs, which have only recently been identified in a few algal species. Previous research indicates that the alga, *Chattonella subsalsa*, possesses a novel NR, NR2-2/2HbN (NR2), which incorporates a 2/2 hemoglobin domain. A second NR (NR3) in *C. subsalsa* lacks the cytochrome b5 (heme-Fe) domain but includes a putative binding motif for 14-3-3 proteins. The expression of *NR2* and *NR3* genes indicates that *NR2* transcript abundance was regulated by light, nitrogen source, and temperature, while *NR3* transcript levels were only regulated by light. Here, we measured total NR activity in *C. subsalsa* and the potential for regulation of NR activity by putative 14-3-3 binding proteins. Results indicate that NR activity in *C. subsalsa* was regulated by light, nitrogen source, and temperature at the translational level. NR activity was also regulated by endogenous rhythm and temperature at the post-translational level, supporting the hypothesis that NR3 is regulated by 14-3-3 binding proteins. Together with a previous report describing the regulation of *NR* gene expression in *C. subsalsa*, results suggest that *C. subsalsa* responds to environmental conditions by differential regulation of NRs at transcriptional, translational, and post-translational levels. This flexibility may provide a competitive advantage for this species in the environment. To date, this is the first report which provides evidence for the potential post-translational regulation of NR by 14-3-3 proteins in algal species and suggests that regulatory mechanisms for NR activity may be shared between plants and some algal species.

## 1. Introduction

Nitrate and ammonium are major nitrogen sources for phytoplankton in the environment (reviewed by Shen et al., 2015; Rezvani et al., 2017; Salbitani and Carfagna, 2021). It has been estimated that 40% of global primary production resulted from the assimilation of these two nitrogen sources by phytoplankton (Malerba et al., 2015 and references therein). These essential nitrogen types also play a role in the stimulation and succession of harmful algal blooms (HABs) and are key factors affecting HAB dynamics (reviewed by Davidson et al., 2012; Wang, 2016; Wurtsbaugh et al., 2019; Coyne et al., 2022). The toxic microalga *Chattonella subsalsa* is among the HAB species implicated in mass fish kills in coastal waters worldwide (reviewed by Imai and Yamaguchi, 2012; Lum et al., 2021). Recent research indicated nitrogen as a major factor affecting *C. subsalsa* growth (Kok et al., 2019), pigmentation (Kok et al., 2019), and competition in natural phytoplankton assemblies (Wang, 2016; Coyne et al., 2022).

For phytoplankton using nitrate as a nitrogen source, nitrate is reduced to nitrite by nitrate reductase (NR), and nitrite is then reduced to ammonium by nitrite reductase (NiR), followed by amino acid assimilation (reviewed by Kumar and Bera, 2020). Nitrate assimilation represents one of the most important redox processes that support 20% of the algal growth in the ocean (reviewed by Bellido-Pedraza et al., 2020). In this process, NR catalyzes the first enzymatic and also rate-limiting step; regulation of this step is essential for the coordination of nitrogen and carbon assimilation in higher plants, algae, and fungi (reviewed by Campbell, 1999; Parker and Armbrust, 2005; Wang et al., 2018). Recent research also indicated NR to be a moonlighting protein that also participates in the production of nitric oxide (reviewed by Sanz-Luque et al., 2015b; Tejada-Jimenez et al., 2019; Bellido-Pedraza et al., 2020, 2022), as well as the conversion of nitric oxide back to nitrate (e.g., Stewart and Coyne, 2011; reviewed by Tejada-Jimenez et al., 2019; Bellido-Pedraza et al., 2020).

It has been well established that the expression and activity of NR are tightly regulated by extra- and intracellular cues (reviewed by Young and Berges, 2016; Wang et al., 2018; Coyne et al., 2022; Yu et al., 2022). The tight regulation of NR is essential for organisms to optimize nitrogen assimilation and prevent the accumulation of toxic compounds, such as nitrite, as well as reactive oxygen and nitrogen species (reviewed by Wang et al., 2018). Nitrogen sources are among the key factors that regulate NR. For instance, nitrate and ammonium have been reported to act oppositely on NR, where NR gene expression and activity can be enhanced by nitrate, while repressed or inhibited by ammonium (reviewed by Sanz-Luque et al., 2015a). When both nitrate and ammonium exist, NR is regulated by the balance between these nitrogen sources (reviewed by Sanz-Luque et al., 2015a; Glibert et al., 2016). The light-to-dark cycle also regulates NR; some algal NRs respond to the light during diurnal changes (e.g., Chow et al., 2004; Wang et al., 2015, 2018), while others change due to an endogenous circadian rhythm (Falcão et al., 2010). Temperature is another factor that may affect NR gene expression and activities in algae (Wang et al., 2018; Zhong et al., 2020). These factors may affect NR independently or synergistically (Parker and Armbrust, 2005). For instance, Parker and Armbrust (2005) demonstrated higher NR gene transcript abundance in diatoms under high light and low temperature conditions, suggesting NR catalyzed nitrogen assimilation may play a role in dissipating excess energy from the photosynthetic light reactions.

In higher plants, NRs are regulated at the post-translational level by the binding of a 14-3-3 protein following the reversible phosphorylation of a serine residue located at the Hinge-1 region (Chi et al., 2015). NR activity can be inhibited by the 14-3-3 binding by obstructing the electron flow between domains through conformational change (Chi et al., 2015). The 14-3-3-mediated NR regulation is crucial for the rapid response of plants to environmental conditions, such as excess nitrate stress (Xu et al., 2016), C/N balance changes (Huarancca Reyes et al., 2018), and light to dark cycles (reviewed by Gao et al., 2014). Although highly conserved in higher plants, 14-3-3 binding motifs were not identified in algal species until recent research (Wang et al., 2018).

**Figure 1:**
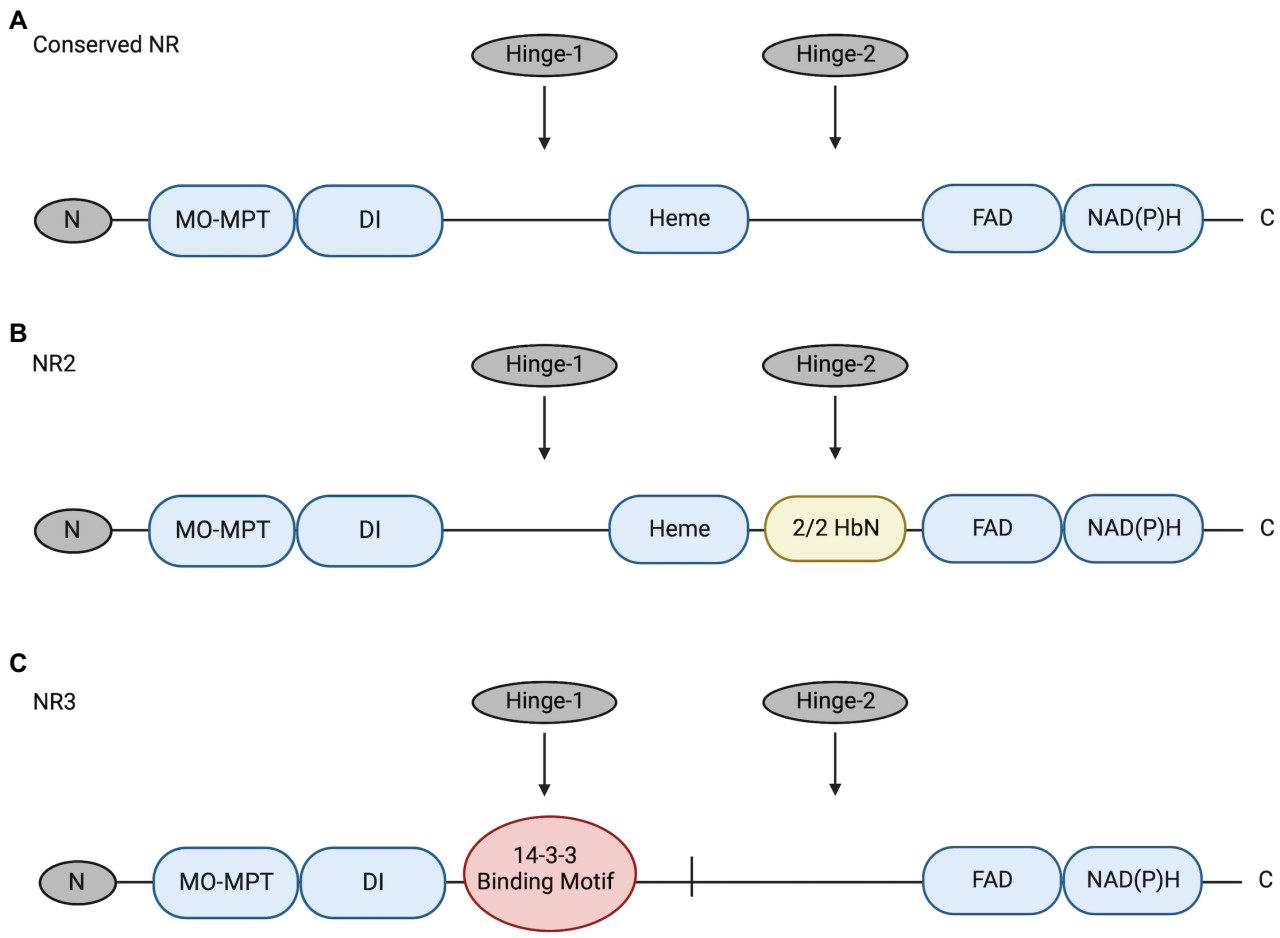
**(A)** Conserved NR structure includes 5 domains (blue; Mo-MPT, DI, Heme, FAD, and NAD[P]H) and 3 regions (grey; N, Hinge-1, and Hinge-2) (Campbell, 1999). **(B)** Structure of NR2 has a 2/2HbN domain (yellow) inserted in the Hinge-2 region. This enzyme was identified in raphidophytes *Heterosigma akashiwo* and *Chattonella subsalsa* (Stewart and Coyne, 2011). **(C)** Structure of the novel NR3 enzyme in *C. subsalsa* that lacks the conserved Heme domain and has a 14-3-3 binding motif (red) in the Hinge-1 region (Wang et al., 2018). The figure was created with Biorender.com and modified from Wang et al. (2018).

NRs are multidomain proteins that are only active as dimers; a 5-domain-and-3-region structure is conserved among NRs of algae, plants, and fungi (reviewed by Campbell, 1999; Chamizo-Ampudia et al., 2017; Wang et al., 2018; Figure 1). These domains include a molybdenum-molybdopterin cofactor domain (Mo-MPT), a dimer interface (DI), a cytochrome b5-binding domain containing Heme-Fe (the Heme-Fe domain), a flavin adenine dinucleotide domain (FAD), and an NAD(P)H domain (reviewed by Campbell, 1999; Chamizo-Ampudia et al., 2017). The three regions include an N-terminal preceding the Mo-MPT domain, a Hinge-1 region between the DI and Heme domains, and a Hinge-2 region between the Heme and FAD domains (reviewed by Campbell, 1999; Chamizo-Ampudia et al., 2017).

Recent research identified novel NRs with alternative structures in *C. subsalsa* and *Heterosigma akashiwo* (Raphidophyceae) (Stewart and Coyne, 2011; Wang et al., 2018; Figure 1). NR2-2/2HbN (referred to hereafter as NR2) identified in *C. subsalsa* and *H. akashiwo* has a 2/2 hemoglobin (2/2Hb) domain inserted in the Hinge-2 region that has been proposed to confer nitric oxide dioxygenase activity to this enzyme (Stewart and Coyne, 2011; Healey et al., 2023). Following research identified another novel NR in *C. subsalsa* (NR3) (Wang et al., 2018). The deduced amino acid sequence of NR3 lacked the 2/2HbN domain found in NR2 as well as the conserved Heme-Fe domain present in all other eukaryotic NRs (Wang et al., 2018). As mentioned earlier, dimerization is necessary for NR to function (reviewed by Chamizo-Ampudia et al., 2017), with a tendency to form tetramers (reviewed by Campbell, 2001). Here, without the conserved Heme-Fe domain, NR3 may form tetramers with NR2 to perform necessary functions as discussed in Wang et al. (2018). Additional sequence analysis also revealed that NR3 included a potential phosphorylation site and a canonical 14-3-3 binding protein motif in the Hinge-1 region (Wang et al., 2018), suggesting the potential for post-translational regulation of this enzyme in a manner similar to plant NRs. Expression of *NR2* and *NR3* in *C. subsalsa* indicated that *NR2* transcript abundance changed in response to light, temperature, and nitrogen source, while *NR3* transcript levels only responded to light (Wang et al., 2018). No research has been conducted to investigate the response of NR enzyme activity in *C. subsalsa* to environmental cues or the potential regulation of putative 14-3-3 protein binding in any algal NRs.

**Figure 2:**
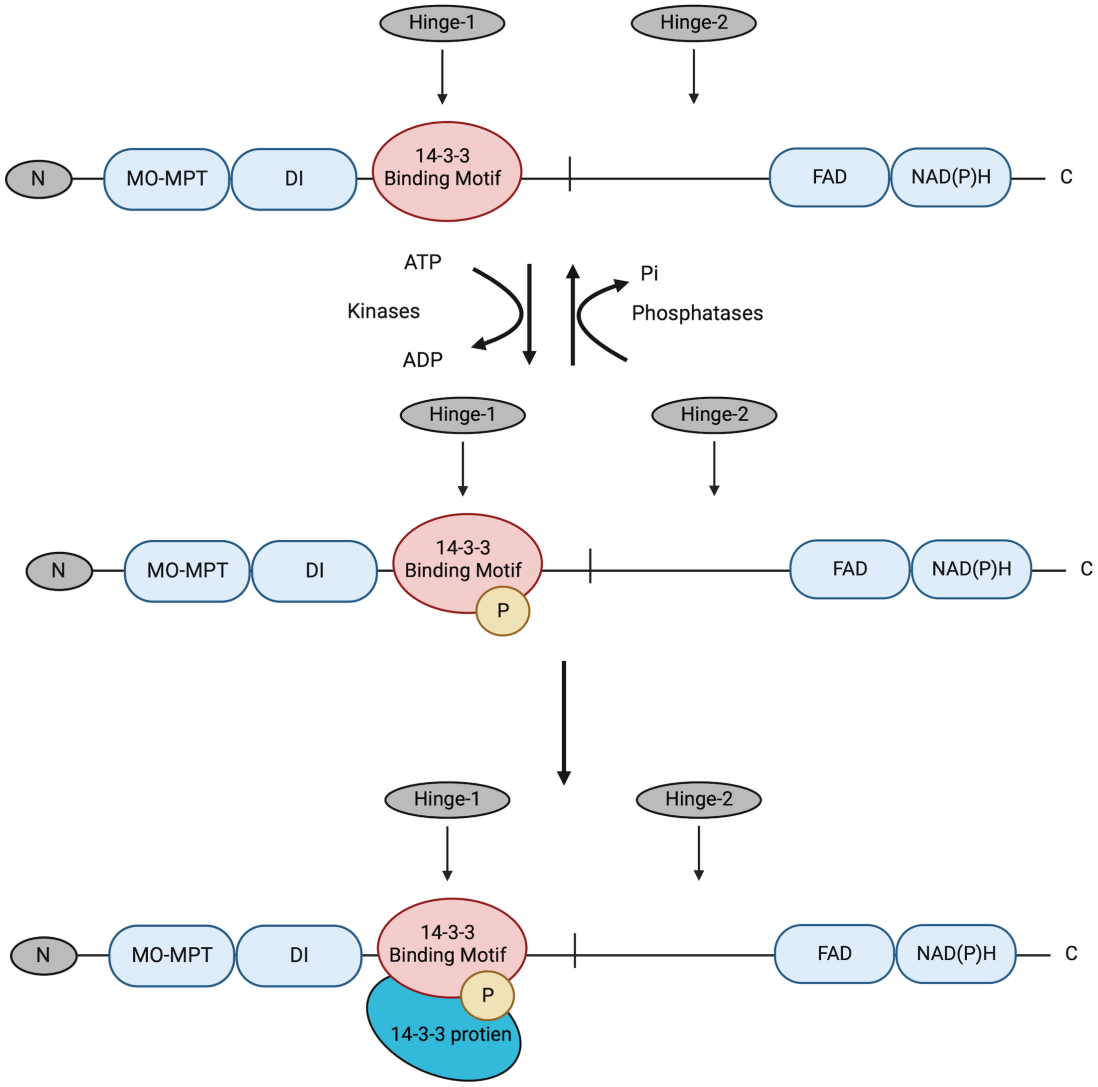
The deduced process for NR3 regulation by reversible phosphorylation and subsequent binding of putative 14-3-3 proteins. The 14-3-3 protein binding motif in the Hinge-1 region is first phosphorylated by kinases; then putative 14-3-3 proteins recognize and bind to the motif for NR3 enzyme activity regulation. This was deduced from the conserved mechanism for NR regulation by 14-3-3 proteins in higher plants (Paul et al., 2012). Pi, phosphate. The figure was created with Biorender.com.

In this study, the post-transcriptional regulation of NR enzyme activity and the potential role of the presumed 14-3-3 proteins in post-translational regulation of NR activity in *C. subsalsa* were examined. NR regulation by 14-3-3 proteins in plants is a two-step process (Figure 2), where NR is first phosphorylated by kinases to allow the subsequent recognition and binding of the 14-3-3 proteins (Paul et al., 2012). Additionally, the binding of 14-3-3 proteins to phosphorylated NR requires divalent cations, such as Mg^2+^, at millimolar concentrations (Athwal and Huber, 2002). The requirement for divalent cations provides a means to evaluate the differential regulation of NR activity by putative 14-3-3 protein binding in cellular homogenates (Mackintosh et al., 1995). In other words, the addition of excess Mg^2+^ would allow the formation of the NR-Ser-P/14-3-3 protein complex in the cellular homogenate. This complex inhibits the activity of the phosphorylated NR, so that the decrease in NR activity is a measure of the remaining non-phosphorylated NR (the actual NR activity) (Mackintosh et al., 1995). On the other hand, adding EDTA to the cellular homogenates would sequester Mg^2+^ through chelation to yield the activity of total NR comprised of both phosphorylated and non-phosphorylated NR (Mackintosh et al., 1995). The difference in activity in the presence and absence of Mg^2+^ provides a measure of NR activity that is inhibited by 14-3-3 protein binding to phosphorylated NR (Mackintosh et al., 1995). Here, changes in NR activity in *C. subsalsa* due to putative 14-3-3 protein binding were investigated in response to light, nitrogen source, and temperature. Results of this study will contribute to our understanding of nitrogen utilization in algal species, and its response to vital environmental cues.

## 2. Materials and methods

### 2.1. Culture conditions

Stock culture of *Chattonella subsalsa* (National Center for Marine Algae and Microbiota, CCMP2191^1^ [accessed on 8/8/2022]) was maintained semi-continuously in exponential growth phase in f/2 medium (without silica) (Guillard and Ryther, 1962), with either f/2 levels of nitrate (882 μM), or 100 μM nitrate or ammonium (only for the nitrogen source experiment below). The salinity of the medium was 20, and the temperature for incubation was 25°C. Stock cultures were maintained without agitation at approximately 100 μE m^−2^ s^−1^ irradiance with a 12:12 h light: dark cycle.

### 2.2. NR activity under different light conditions

#### 2.2.1. Long-term light experiment

*C. subsalsa* was cultured as above with f/2 levels of nitrate as the nitrogen source. To test for changes in activity after acclimation to the light regime, replicate cultures (*N* = 3; culture volume = 500 ml) were exposed to either a 12:12 h light: dark cycle or to constant light at 100 μmol photons m^−2^ s^−1^ for 24 h before the initial sampling. Cultures in constant light remained so for the entire sampling period. Samples were collected at five time points to cover a wide range of timing before and after the light changes for cultures under the light: dark cycle: 1 h before lights on (1:00), 1 h after lights on (3:00), 6 h after lights on (8:00), 1 h after dark (15:00), and 6 h after dark (20:00). Samples from cultures under the constant light were also collected at the same time-points to compare with those under the light to dark cycle. At each time point, samples of 50 ml were collected and centrifuged at 6000 RPM for 5 min. The supernatant was discarded, the cell pellet was immediately frozen in liquid nitrogen and stored at −80°C until analysis (see below).

#### 2.2.2. Short-term light experiment

To test for short-term regulation of NR activity, *C. subsalsa* was cultured as above in f/2 medium with a 12:12 light: dark cycle. At 6 h after lights on, replicate cultures (*N* = 3; culture volume = 50 ml) were transferred to the dark. Controls (*N* = 3) remained in the light. Samples were collected at 15 min after transferring to the dark and processed as above for the NR activity assay described below.

### 2.3. NR activity with different nitrogen sources

*C. subsalsa* was cultured without agitation in f/2 medium with either 100 μM NO_3_^−^ or 100 μM NH_4_^+^ as the nitrogen source (*N* = 3; culture volume > 100 ml). The temperature and light conditions for these cultures were described above as for the stock cultures. The semi-continuous cultures were acclimated to growth with each nitrogen type and maintained in exponential growth before harvesting. Cultures were harvested at 6 h after the start of the light cycle and processed as described above for the NR activity assay.

### 2.4. NR activity at different temperatures

#### 2.4.1. Long-term temperature acclimation experiment

To test for changes in activity after acclimation to temperature, *C. subsalsa* was cultured without agitation in f/2 medium with nitrate (f/2 level) at 28°C, 25°C, and 18°C (N = 3; culture volume > 100 ml). The light intensity was the same as the stock cultures. Semi-continuous cultures were maintained in exponential growth at each temperature condition for 2 months before harvesting. Samples were collected at 6 h after the start of the light cycle as described above for NR activity analysis.

#### 2.4.2. Short-term temperature change experiment

To test for short-term regulation of NR activity, *C. subsalsa* was cultured as above in f/2 medium at 25°C. At 6 h after lights on, replicate cultures (*N* = 3; culture volume = 50 ml) were transferred to a water bath at 8°C. Controls (*N* = 3) remained at 25°C. To be noted, 8°C is below *C. subsalsa*’s growth temperature (10 to 30°C; Zhang et al., 2006) and was used here to assess its NR activity under cold stress. Samples were collected at 15 min after transferring to the cold and processed for the NR activity assay as described above. Only total NR activity was assayed for the controls.

### 2.5. NR activity assay

The NR activity assay was optimized for *C. subsalsa* according to the methods published by Berges and Harrison (1995). The assay was optimized for homogenization methods (grinding vs. sonication), extraction buffers (KPi [potassium phosphate] buffer, BTP [Bis-tris propane] buffer, TAPS {[tris(hydroxymethyl)methylamino] propanesulfonic acid}) buffer, EPPS [HEPPS] buffer, Tris [Tris(hydroxyl-methyl)aminomethane] buffer, and MOPS [3-morpholinopropane-1-sulfonic acid] buffer), stabilizing additives (BSA [Bovine Serum Albumin], DTT [dithiothreitol], and BSA plus DTT), specificity for NADH versus NADPH, as well as assay temperatures and pH. Ca^2+^ was also tested as an alternative divalent cation for Mg^2+^ to measure the activity of non-phosphorylated NR but did not improve the assay. The optimized protocol included lysing cells by sonication, followed by extraction in 200 mM KPi buffer [200 mM KH_2_PO_4_, 2.5% (w/v) KOH], pH 7.9, using NADH as a reductant, and with no stabilizing additives. Mg^2+^ was used as the divalent cation when required in the assay below. The assay was conducted at 25°C.

The frozen cell pellet was resuspended in 1 ml 200 mM KPi extraction buffer (pH 7.9) on ice, homogenized by sonication, and centrifuged to clarify at 4°C. The supernatants were assayed immediately. The homogenate was divided to evaluate total NR activity (with EDTA to chelate endogenous Mg^2+^) or NR activity in the presence of excess Mg^2+^ to measure non-phosphorylated NR (Berges and Harrison, 1995; Nemie-Feyissa et al., 2013). The optimized assay consisted of 100 μl extract, an equal volume of assay buffer (500 mM KPi, pH 7.9, amended with 2 mM EDTA or 6 mM MgCl_2_), 0.2 mM NADH (Sigma Chem. Co., St. Louis, MO) and 10 mM KNO_3_ as substrate. Sterile water was added to the negative control reaction in place of KNO_3_. The reaction was incubated for 30 min at room temperature in the dark. Zinc acetate (12.5 μl of 1 M stock) was added to stop the reaction. The samples were centrifuged, and the supernatants were used below.

The concentration of nitrite product in the supernatants was measured colorimetrically. Ten microliters of 750 μM N-Methylphenazonium methyl sulfate (Sigma–Aldrich Fluka, Buchs SG, Switzerland) were added to 200 μl supernatants. The reaction was incubated in the dark for 20 min, followed by the addition of 12.5 μl 5 M HCl and 125 μl 58 mM Sulfanilamide (Sigma Chem. Co.). After an additional 5 min of incubation in the dark, 125 μl 4 mM N-(1-Naphthyl) ethylenediamine dihydrochloride (Sigma Chem. Co.) was added to the reaction. The samples were incubated for 10 min and then were aliquoted into 96 well plates. The absorbance was measured at 540 nm on a microplate reader (BMG Labtech, Ortenberg, Germany). NR activity was normalized to the protein content of the cellular homogenate as measured using the Pierce BCA Protein Assay Kit (Pierce, Rockford, IL, United States).

### 2.6. Statistical analysis

Repeated measures ANOVA was used to test if the activity of total NR and non-phosphorylated NR changed significantly over time in the long-term light experiment. If the change was significant, then a paired t-test was conducted to analyze the significance of the differences between the values at each time point. Two sample t-test was used to test the significance of the difference between total or non-phosphorylated NR activity in the light to dark cycle compared to constant light at each time point in the long-term light experiment. Two sample t-test was also used to assess the significance of difference between total or non-phosphorylated NR activity comparing the light conditions (light vs. dark) in the short-term experiment.

One-way ANOVA was used to test if different temperatures had a significant effect on the total or non-phosphorylated NR activity in the long-term temperature experiment. If the effect was significant, then Tukey’s HSD test was used to analyze the difference between values in all possible pairs of groups. Two sample t-test was used to assess the significance of difference between the total NR activity comparing different temperatures in the short-term experiment.

Additionally, a paired t-test was used to test if there was a significant difference between total and non-phosphorylated NR in all experiments under each of the light, nitrogen, and temperature conditions. A significance level of 0.05 was used in all tests. All statistical analyses were performed in R (V.3.2.4 Revised, R Core Team 2016).

## 3. Results

### 3.1. Diel activity of NR under different light conditions

#### 3.1.1. Long-term light experiment

**Figure 3:**
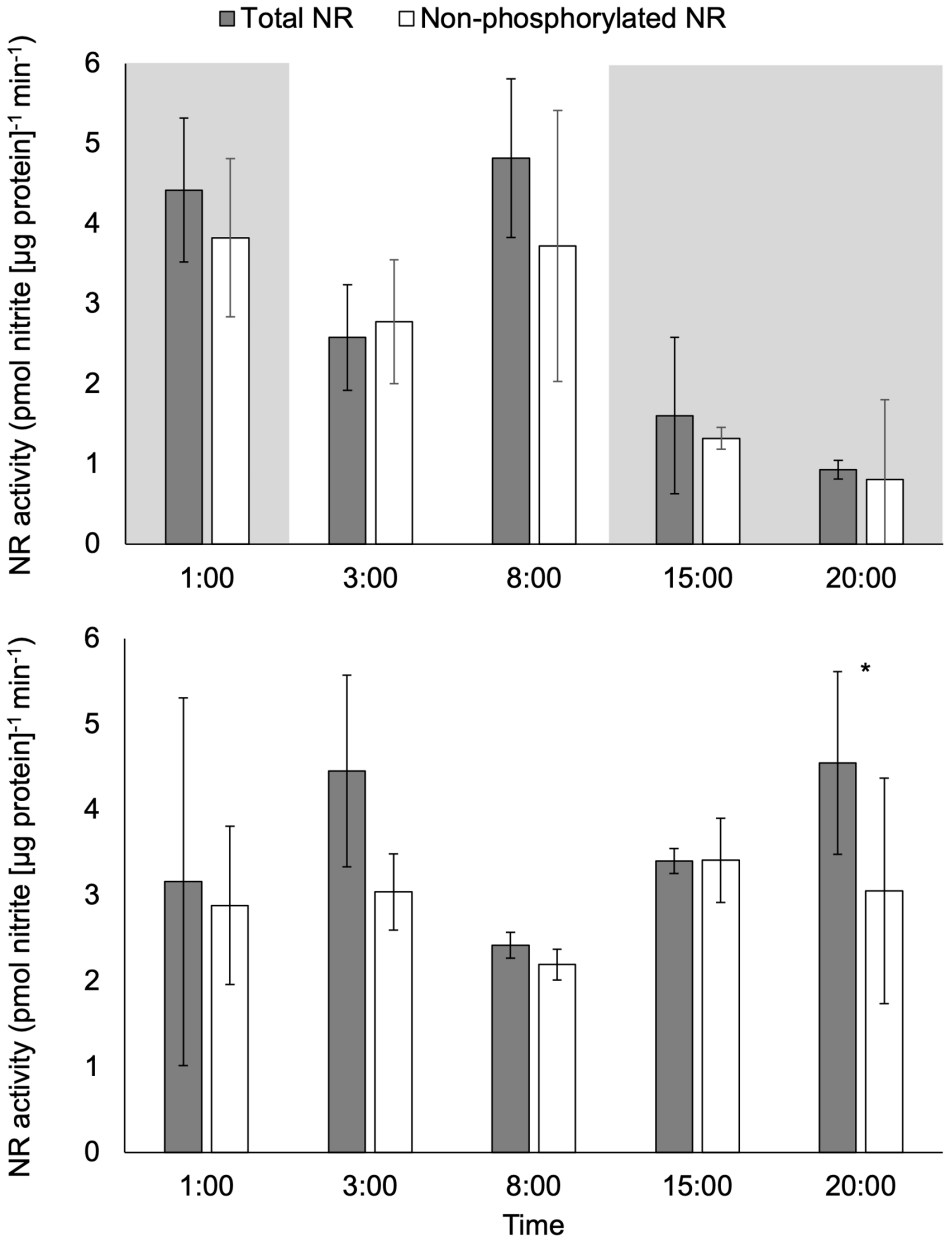
Activity (pmol nitrite [μg protein]^−1^ min^−1^) of total NR and non-phosphorylated NR in *C. subsalsa* cultured in a 12:12 h light: dark cycle (upper panel) or constant light (lower panel) in the long-term light experiment. The portion with shade indicates the time that was in dark for algae cultured with the light: dark cycle. Samples were taken at 1 h before lights on (1:00), 1 h after lights on (3:00), 6 h after lights on (8:00), 1 h after dark (15:00), and 6 h after dark (20:00). Asterisk (*) reveals the significant difference in NR activity between total and non-phosphorylated NR, indicating the regulation by reversible phosphorylation and the subsequent binding of putative 14-3-3 proteins, at the indicated time point (*p* < 0.05). Error bars are standard deviation of three biological replicates.

There was a significant difference in activity between total NR activity (assay conducted with EDTA) and non-phosphorylated NR activity (assay conducted with excess Mg^2+^) in samples under constant light at 20:00 (*p* < 0.05; Figure 3). No significant difference between total NR and non-phosphorylated NR activity was observed at any other time points under constant light or at any time point in the light:dark treatment (*p* > 0.05).

Total NR activity decreased significantly over time in the light:dark treatment (*p* < 0.05) from mid-day (8:00) to 6 h after dark (20:00, *p* < 0.05; Figure 3). There were no significant changes over time in total NR activity under constant light (*p* > 0.05). Comparison of total NR activity in light:dark treatment to constant light revealed significantly higher activity in the light:dark treatment at 8:00 (6 h after lights on) and significantly lower activity at 20:00 (6 h after dark, *p* < 0.05).

The activity of non-phosphorylated NR for the light:dark treatment was significantly higher at 1 h (1:00) before lights on than at 1 h after dark (15:00 *p* < 0.05; Figure 3). There were no significant changes over time in the activity of non-phosphorylated NR under constant light (*p* > 0.05). Non-phosphorylated NR activity was significantly lower at 1 h after dark (15:00) in the light:dark treatment than non-phosphorylated NR activity from the same time point in constant light treatment (*p* < 0.05).

#### 3.1.2. Short-term light experiment

In the short-term experiment, there was no significant difference in total or non-phosphorylated NR activity between samples in light vs. those transferred to dark for 15 min (*p* > 0.05; Supplementary Figure S1). Additionally, there was no significant difference in total and non-phosphorylated NR activity within samples for either light condition (*p* > 0.05; Supplementary Figure S1).

### 3.2. NR activity during growth with different nitrogen sources

Only the treatments cultured with NO_3_^−^ exhibited NR activity (Supplementary Figure S2), while activity in treatments with NH_4_^+^ was below detection. For the nitrate treatments, there was no significant difference between the total and non-phosphorylated NR activity (*p* > 0.05).

### 3.3. NR activity when growing at different temperatures

#### 3.3.1. Long-term temperature acclimation experiment

**Figure 4:**
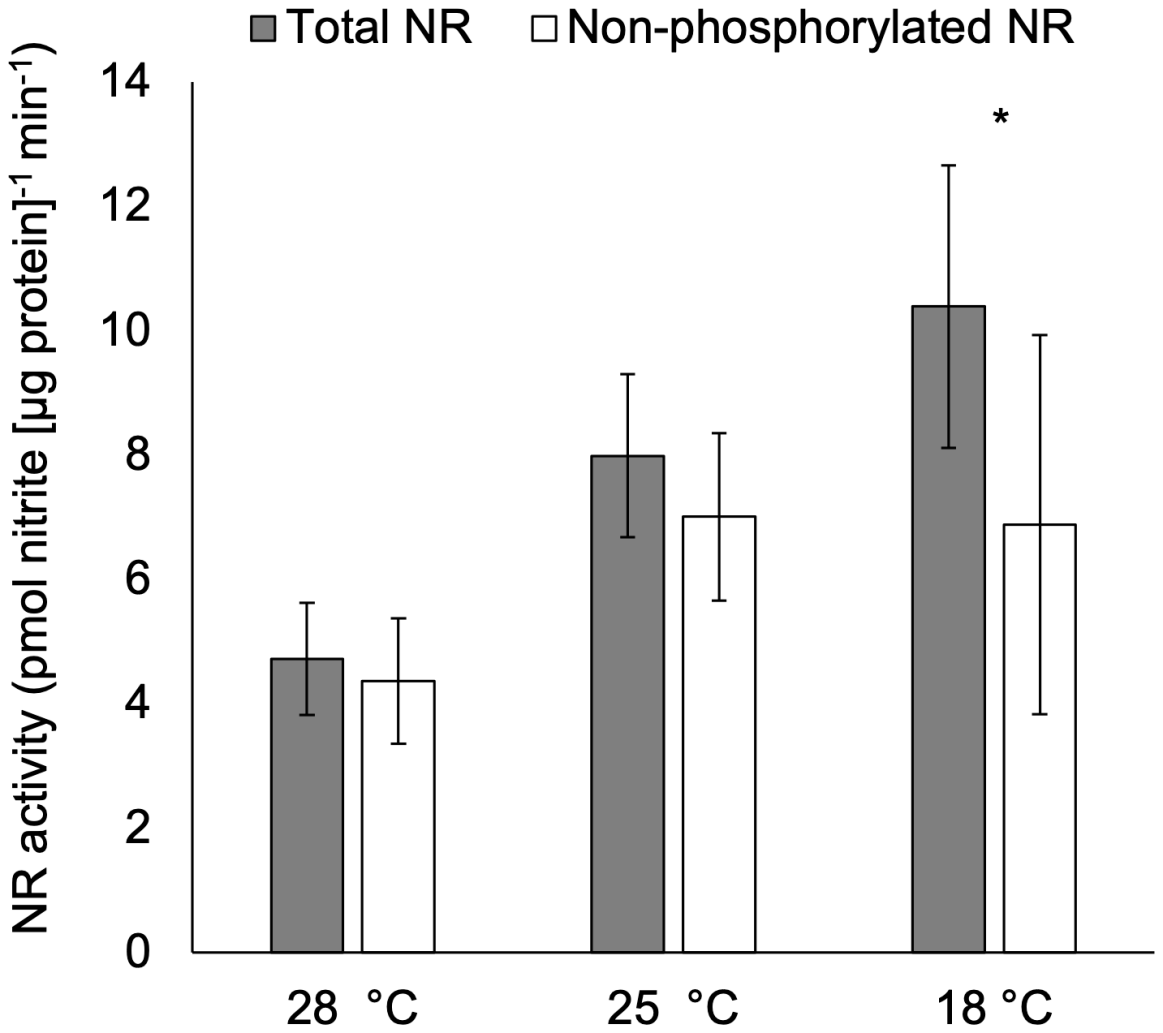
Activity (pmol nitrite [μg protein]^−1^ min^−1^) of total NR and non-phosphorylated NR in *C. subsalsa* acclimated to different temperatures (28°C, 25°C, and 18°C) in the long-term temperature experiment. Samples for NR activity assay were collected at 6 h after lights on. Asterisk (*) reveals the significant difference in NR activity between total and non-phosphorylated NR, indicating the regulation by reversible phosphorylation and the subsequent binding of putative 14-3-3 proteins, at the indicated temperature (*p* < 0.05). Error bars are standard deviation of three biological replicates.

After acclimation to temperatures ranging from 18 ° to 28°C, total NR activity was significantly higher in cultures at 18°C compared to those at 28°C (*p* < 0.05; Figure 4), but no significant difference was observed in total NR activities in treatments cultured at 25°C compared to those at 18°C or 28°C (*p* > 0.05; Figure 4). Furthermore, non-phosphorylated NR activity was significantly lower than total NR activity in cultures acclimated to 18°C (*p* < 0.05), but not in the other two temperature treatments (*p* > 0.05).

#### 3.3.2. Short-term temperature change experiment

**Figure 5:**
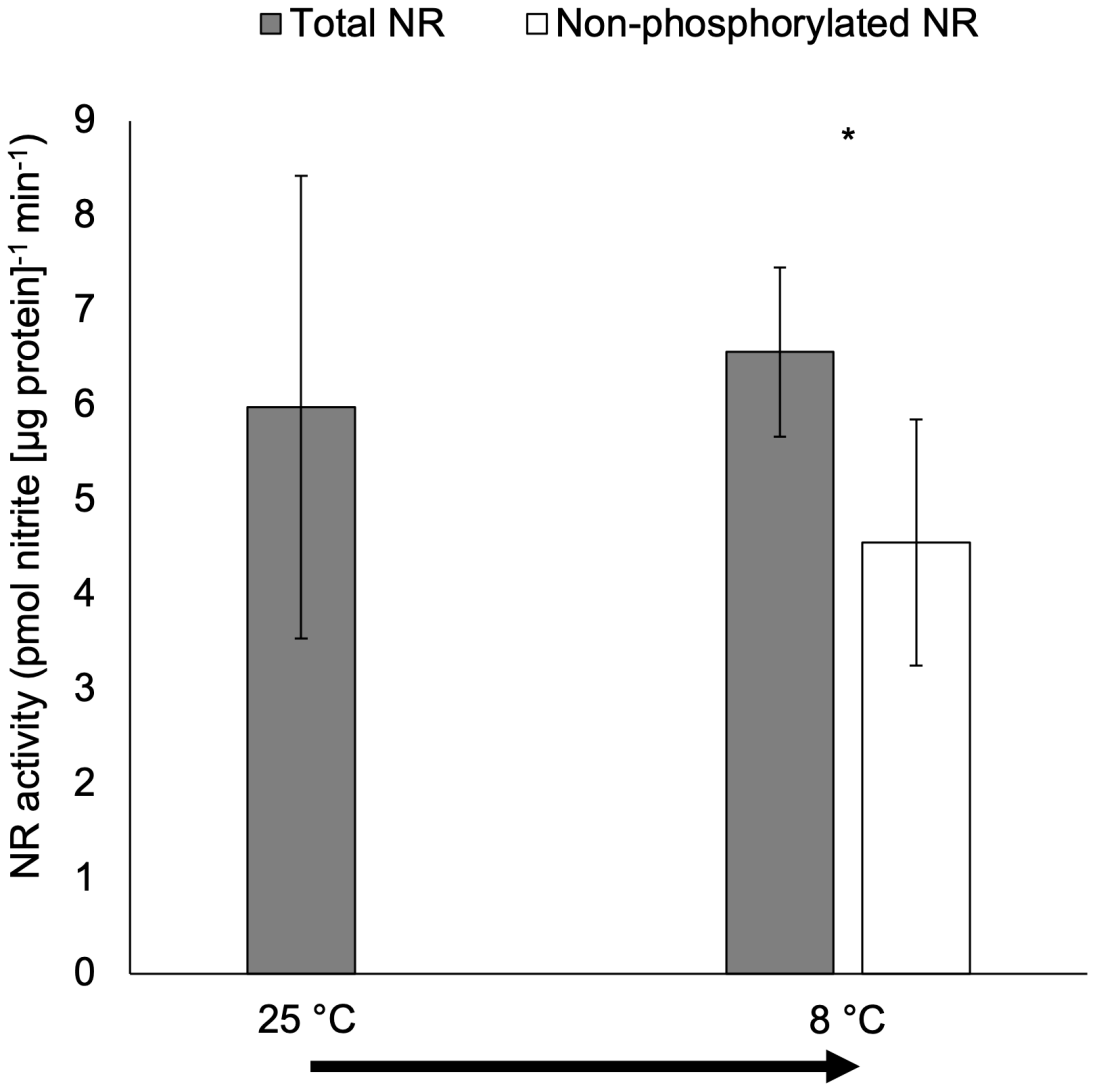
Activity (pmol nitrite [μg protein]^−1^ min^−1^) of total NR and non-phosphorylated NR in the short-term temperature experiment when the culture was transferred from 25°C to 8°C (15-min incubation after the transfer; only total NR activity was measured for the culture under 25°C). The arrow indicates the short-term temperature change. Samples for NR activity assay were collected at 6 h after lights on. Asterisk (*) reveals the significant difference in NR activity between total and non-phosphorylated NR, indicating the regulation by reversible phosphorylation and the subsequent binding of putative 14-3-3 proteins, at the indicated temperature (*p* < 0.05). Error bars are standard deviation of three biological replicates.

There were no significant differences in total NR activity for cultures moved from 25°C to 8°C in the short-term temperature manipulation experiment (*p* > 0.05; Figure 5). However, non-phosphorylated NR activity was significantly lower than total NR activity for cultures moved from 25°C to 8°C (*p* < 0.05).

## 4. Discussion

This study aimed to investigate post-transcriptional (translational and/or post-translational) regulation of nitrate reductase enzymes (NR) in the harmful alga *C. subsalsa*. In contrast to NR1 and NR2 in *H. akashiwo*, which are identical except for the insertion of a 2/2HbN domain in NR2 (Coyne, 2010; Stewart and Coyne, 2011), analysis of NR amino acid sequences in *C. subsalsa* shows that NR3 was distinct from NR2 (Wang et al., 2018; Figure 1). *C. subsalsa* NR3 contained a potential phosphorylation site and a canonical 14-3-3 protein binding motif in the Hinge-1 region similar to those found in plant NRs (Wang et al., 2018), suggesting that this enzyme may be regulated by 14-3-3 protein binding (Figure 2). Here, the maximum, or total, NR enzyme activity (phosphorylated and non-phosphorylated) was compared to non-phosphorylated NR as a measure of “actual” or uninhibited activity within the cell. Differences between total and non-phosphorylated NR activity indicate a response to environmental conditions or nutrient status regulated by reversible phosphorylation and putative binding of 14-3-3 proteins. In this investigation, responses to light regime, nutrient source, and temperature were evaluated in order to identify factors involved in post-translational regulation of NR activity by putative 14-3-3 protein binding.

The previous study on the gene expression profile of *NR2* and *NR3* in *C. subsalsa* under light: dark cycle vs. constant light demonstrated that the gene expression of both enzymes was regulated by light rather than the circadian clock (Wang et al., 2018). A similar pattern was observed for the activity of total and non-phosphorylated NR in *C. subsalsa*, with activity responding to the light regime and not regulated by an endogenous diel rhythm (Figure 3). However, NR activity was decoupled from gene expression in *C. subsalsa*, with the highest activity near mid-day (Figure 3 upper panel), following the gene expression maximum in the early morning (Wang et al., 2018). Decoupling of expression and activity has been observed in other plants and algae (Chow et al., 2004; Lea et al., 2006; Falcão et al., 2010), in which the highest levels of NR activity matched the high photosynthetic rates accompanying the light phase (e.g., Granbom et al., 2004; Lea et al., 2006; Young et al., 2007). The similar rhythm of photosynthesis and nitrate assimilation is likely due to a combination of the requirement for ATP and reduced ferredoxin generated by photosynthesis for nitrite reduction, while NR serves as a crucial sink for excess reductant produced by the light reactions of photosynthesis (reviewed by Chow et al., 2004; Sanz-Luque et al., 2015a; Glibert et al., 2016).

There were no significant differences between the activity of total and non-phosphorylated NR in response to a 12:12 light: dark cycle (Figure 3), or in the short-term light shift experiment (Supplementary Figure S1), indicating that light-driven changes in NR activity were not regulated post-translationally by putative 14-3-3 protein binding at time points investigated here. These results differed from the reports on plant NRs, where these enzymes are transiently inactivated by binding of 14-3-3 proteins responding to a change in light intensity (Lea et al., 2006; Nemie-Feyissa et al., 2013). It is likely that other mechanisms played a role in regulating NR activity in *C. subsalsa* in response to changes in the light regime. For instance, research in plants demonstrated NR phosphorylation by mitogen-activated protein kinases (MAPKs) play a role in the regulation of its activity (Wang et al., 2010), and recent research suggested MAPK kinase kinases (MAPKKKs) in the green alga *Chlamydomonas reinhardtii* may also be involved in nitrogen assimilation (Gomez-Osuna et al., 2020). The role of alternative mechanisms involved in the regulation of *C. subsalsa* NR activity is unknown and requires future research.

In higher plants, 14-3-3 proteins modulate the circadian activation of hydraulic conductivity, which is essential for plants to synchronize water supply to optimize growth with diurnal cycles (Prado et al., 2019). This regulatory mechanism is also evident in studies demonstrating a post-translational oscillator and protein phosphorylation involved in the circadian-clock-mediated cellular processes in cyanobacteria (Chang et al., 2015; Tseng et al., 2017; Brenna and Albrecht, 2020). In the research conducted here, the significant difference in total vs. non-phosphorylated NR activity at 6 h after dark (20:00) in cultures maintained at constant light suggests that putative 14-3-3 binding of NR3 is influenced by an endogenous rhythm that is not affected by light regime (Figure 3).

NR activity in *C. subsalsa* was responsive to nitrogen sources, with measurable NR activity in treatments cultured with nitrate (Supplementary Figure S2) but no detectable activity in ammonium treatments, demonstrating the translational regulation of NR by nitrogen sources in this species. This is in contrast to previous research showing constitutive expression of *NR2* and *NR3* transcripts in cultures of *C. subsalsa* supplied only with ammonium (Wang et al., 2018), and suggests feedback inhibition by the downstream products of nitrogen assimilation at the level of translation (Ali et al., 2007). These results are consistent with reports on other algal species, where NR activity is strongly suppressed by high concentrations of ammonium (Young et al., 2007; Chow and de Oliveira, 2008). Additionally, there was no indication for putative 14-3-3 binding involved in post-translational regulation in *C. subsalsa* cultured with nitrate here. To be noted, the nitrate concentration used in this study (100 μM) was much higher than concentrations typically measured in the environment. It has been well established that in higher plants, NRs are regulated at the post-translational level with reversible phosphorylation by the relative proportions of nitrate to ammonium, as well as the carbon to nitrate or ammonium balances (Engelsberger and Schulze, 2012; Huarancca Reyes et al., 2018). For instance, in *Arabidopsis*, phosphorylation levels of NR were higher in cultures resupplied with ammonium compared to nitrate after nitrogen starvation (Engelsberger and Schulze, 2012), and NR was inhibited by reversible phosphorylation and subsequent binding of 14-3-3 proteins when treated with elevated glucose and nitrate or ammonium as the nitrogen source (Huarancca Reyes et al., 2018). Additional research will further our understanding of NR regulation in *C. subsalsa* exposed to more environmentally relevant nitrate and ammonium concentrations.

The role of protein phosphorylation and 14-3-3 protein binding in regulating NR activities in response to temperature has also been reported in higher plants. For example, NR was activated by low temperatures through protein dephosphorylation and the subsequent dissociation of 14-3-3 binding proteins in leaves from winter wheat (*Triticum aestivum* L. cv. Sadovo-1) (Yaneva et al., 2002). Reports indicate that 14-3-3 proteins also play other roles in temperature-mediated regulation; these proteins regulate freezing tolerance and cold acclimation in higher plants by controlling cold-induced gene expression (Catalá et al., 2014) and protect animal cells from heat stress by dissolving thermal-aggregated proteins (Yano et al., 2006). Little is known, however, about the temperature-mediated regulatory mechanisms involved in enzyme activity in unicellular algae. In *C. subsalsa*, a significant difference between total and non-phosphorylated NR was observed at lower temperatures in both the long- (18°C) and short-term (8°C) experiments in this study (Figures 4, 5), providing the strongest evidence for post-translational regulation of NR activity involving reversible phosphorylation and putative 14-3-3 protein binding.

Elevated total NR activity at lower temperatures observed in the long-term temperature acclimation experiment (Figure 4) could be partially driven by regulation at the transcriptional level, where expression of *NR2* in *C. subsalsa* was highest in cultures acclimated to lower temperatures (Wang et al., 2018). Higher NR activity at lower temperatures was also observed in other studies and suggested that increased NR activity may act to dissipate excess energy generated under high-light and low-temperature conditions (Parker and Armbrust, 2005). Light conditions here, however, were not excessive for *C. subsalsa* (100 μE m^−2^ s^−1^) (Zhang et al., 2006). This may have contributed to the fine-tuning of post-translational regulation of NR activity at 18°C by putative 14-3-3 proteins. Evidence presented here and in Wang et al. (2018) suggests that the up-regulation of transcription (Wang et al., 2018) and the higher total activity of NR at lower temperatures (Figure 4) after long-term acclimation were modulated by post-translational mechanisms that occurred over a short period of time. That is, the observed significant difference in total vs. non-phosphorylated NR within 15 min after the shift from 25°C to 8°C (Figure 5) persisted after long-term acclimation to low temperature (Figure 4). Under low light, low temperature conditions, results presented here suggest post-translational mechanisms involving reversible phosphorylation and putative 14-3-3 protein binding to inhibit NR activity. This inhibition in activity occurred in spite of higher transcript abundance (Wang et al., 2018) and total NR activity, and resulted in no significant differences in non-phosphorylated (uninhibited) NR enzyme activity over the temperature range tested here (Figure 4).

In contrast to the long-term temperature acclimation experiment, there was no increase in the activity of total NR when cultures were shifted to a lower temperature during the short-term experiment (Figure 5), demonstrating that mechanisms involved in the translational regulation of NRs in *C. subsalsa* responding to temperatures require a longer period of cold stress. Together, these results indicate that the (relatively) slower translational response results in elevated total NR activity at lower temperatures after long-term acclimation, but that putative reversible phosphorylation and 14-3-3 protein binding can rapidly modulate NR activity in response to other environmental factors.

In conclusion, the results of this study demonstrated translational and post-translational regulation of NR enzymes in *C. subsalsa* by different environmental factors. Total NR activity was regulated at the level of translation by light, nitrogen source, and temperature. At the post-translational level, evidence was presented supporting the regulation of NR activity in *C. subsalsa* through the reversible phosphorylation and subsequent binding of 14-3-3 proteins under constant light and low-temperature conditions. The ability of *C. subsalsa* to differentially regulate NRs at multiple levels (transcriptional, translational, and post-translational levels) may allow this species to fine tune nitrogen assimilation with photosynthetic activities in response to environmental cues. To date, this is the first report of NR regulation at the post-translational level involving reversible phosphorylation and subsequent binding of putative 14-3-3 proteins in algal species and suggests that regulatory mechanisms for NR activity may be shared between plants and some algal species.

## Data availability statement

The raw data supporting the conclusions of this article will be made available by the authors, without undue reservation.

## Author contributions

YW conducted the research, analyzed the data, and wrote the manuscript. GJ conducted the research and contributed to data analysis. AP processed the samples. KC conceived of and supervised the project and contributed to manuscript preparation. All authors contributed to the article and approved the submitted version.

## Funding

This research was funded in part by the Gordon and Betty Moore Foundation through grant #2637 to the National Center for Genome Resources. This research was also funded by Delaware Sea Grant College Program (grant #R/SCD-2 to KC). Additional funding was provided through the National Oceanic and Atmospheric Administration National Center for Coastal Ocean Science (NCCOS) Ecology and Oceanography of Harmful Algal Blooms (ECOHAB) program (grant # NA18NOS4780165 to KJC); publication #ECO1042.

## Conflict of interest

The authors declare that the research was conducted in the absence of any commercial or financial relationships that could be construed as a potential conflict of interest.

## Publisher’s note

All claims expressed in this article are solely those of the authors and do not necessarily represent those of their affiliated organizations, or those of the publisher, the editors and the reviewers. Any product that may be evaluated in this article, or claim that may be made by its manufacturer, is not guaranteed or endorsed by the publisher.

## Footnotes

^1^https://ncma.bigelow.org
