# Supplementary material for: Nitrate reductase enzymes in alga Chattonella subsalsa are regulated by environmental cues at the translational and post-translational levels
Source: Front Microbiol. 2023 Mar 2;14:1059074. doi: 10.3389/fmicb.2023.1059074 (PMC10018130; doi:10.3389/fmicb.2023.1059074)
Supplement: Supplementary file 1 [file Data_Sheet_1.docx]

**Supplementary Material**

**Supplementary Figure 1.** Activity (pmol nitrite [μg protein]^-1^ min^-1^) of total NR and non-phosphorylated NR in *Chattonella subsalsa* in the short-term light experiment. *C. subsalsa* was cultured in f/2 medium with a 12:12 light: dark cycle under 25 °C. At 6 hours after lights on, replicate cultures were transferred to the dark (the “Dark” group; N=3). Controls (the “Light” group; N=3) remained in the light. Samples were collected 15 minutes after transferring to the dark. Error bars are standard deviation of three biological replicates. No significant differences were observed between the activities of total and non-phosphorylated NR at either light conditions or between total or non-phosphorylated NR activities comparing light vs. dark (p>0.05).

**Supplementary Figure 2.** Activity (pmol nitrite [μg protein]^-1^ min^-1^) of total NR and non-phosphorylated NR in *Chattonella subsalsa* cultured with 100 μM nitrate as the sole nitrogen source in the nitrogen experiment. NR activity of *C. subsalsa* cultured with 100 μM ammonium was below detection limits. The cultures were maintained under a 12:12 light: dark cycle and 25 °C. Cultures were harvested at 6 hours after the start of the light cycle. Error bars are standard deviation of three biological replicates. No significant differences were observed between the activities of total and non-phosphorylated NR (p>0.05).
